# Extensive Subcutaneous Emphysema Following Thoracoscopic Lung Wedge Resection

**DOI:** 10.7759/cureus.100202

**Published:** 2025-12-27

**Authors:** Khuloud H Alnuaimi, Reem F Almerri, Mouza S Alneyadi, Zayed J Alhammadi

**Affiliations:** 1 Emergency Medicine, Sheikh Tahnoon Bin Mohammed Medical City (STMC), Al-Ain, ARE; 2 Emergency Medicine, Tawam Hospital, Al-Ain, ARE

**Keywords:** airway compromise, blowhole incision, lung wedge resection, postop subcutaneous emphysema, subcutaneous drain

## Abstract

Subcutaneous emphysema (SCE) is a recognized but uncommon complication of thoracic surgery. While it is often self-limiting, severe cases may progress rapidly and compromise the airway. We report the case of a 71-year-old man with interstitial lung disease who developed extensive SCE two weeks after thoracoscopic wedge resection of the lung. He presented with progressive facial and neck swelling, hoarseness, and dyspnea. On examination, he was tachypneic with extensive subcutaneous crepitus. Imaging confirmed widespread emphysema extending through the thoracic wall and soft tissues with associated pneumomediastinum. Bilateral "blowhole" incisions were performed with wet dressings and manual decompression; however, his condition deteriorated, and he developed stridor, requiring emergency intubation in the operating room. Heliox was considered but unavailable. Definitive improvement was achieved after placement of a 24 Fr subcutaneous drain connected to continuous suction. The patient was gradually weaned from ventilatory support and discharged in stable condition. This case underscores the importance of early recognition, escalation of therapy when conservative measures fail, and the potential role of subcutaneous drainage in severe postoperative SCE.

## Introduction

Subcutaneous emphysema (SCE) refers to the presence of air within the subcutaneous tissues, most commonly arising as a complication of thoracic procedures, trauma, or infections. While usually benign, extensive SCE can cause airway compromise, dysphagia, and respiratory distress. Reported incidence after thoracic surgery ranges from 1% to 6% [1,2]. There are no standardized management guidelines as every case is different in its etiology, progression of SCE (hence, has to be managed depending on the big picture scenario), and is based on surgeon preference, ranging from conservative observation to decompressive techniques [2,3].

We report a case of extensive SCE developing two weeks after thoracoscopic lung wedge resection. The patient deteriorated despite initial conservative decompression and ultimately required emergency intubation and placement of a subcutaneous drain with continuous suction. This case underscores the importance of early recognition, vigilant monitoring, and timely escalation of therapy when conservative measures fail, emphasizing the role of multidisciplinary management in preventing morbidity.

## Case presentation

A 71-year-old Sudanese man with a history of interstitial lung disease was admitted for acute pneumonitis and underwent thoracoscopic right lung wedge resection and pleural biopsy. Three specimens were taken from different locations, including the lower lobe wedge (2.2 x 2 x 0.5 cm), middle lobe wedge (4.5 x 2 x 0.5cm), and pleural tissue (1 x 1 x 0.3 cm). Tissue stapling was performed using an Echelon Powered Stapler. A 24 Fr chest tube was inserted and secured to the skin with 0.2 silk sutures, and it was connected to the Thopaz chest drain system at -20 cm H₂O with no air leak. The pathology report from the samples revealed a non-necrotizing granulomatous lung disease.

**Figure 1:**
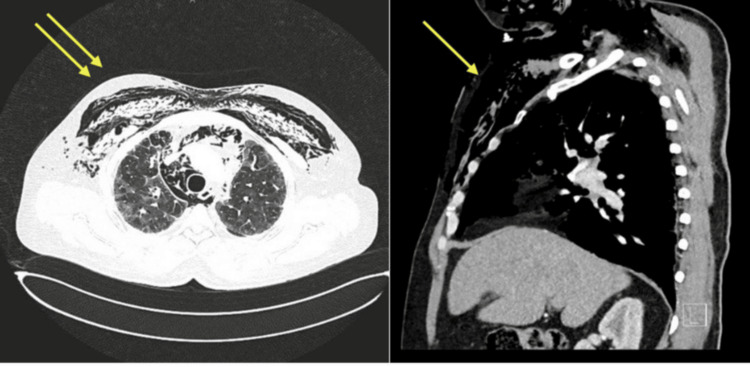
(A, B) CT chest showing extensive subcutaneous emphysema (yellow arrows) tracking through the chest wall and soft tissues (axial and sagittal views).

**Figure 2:**
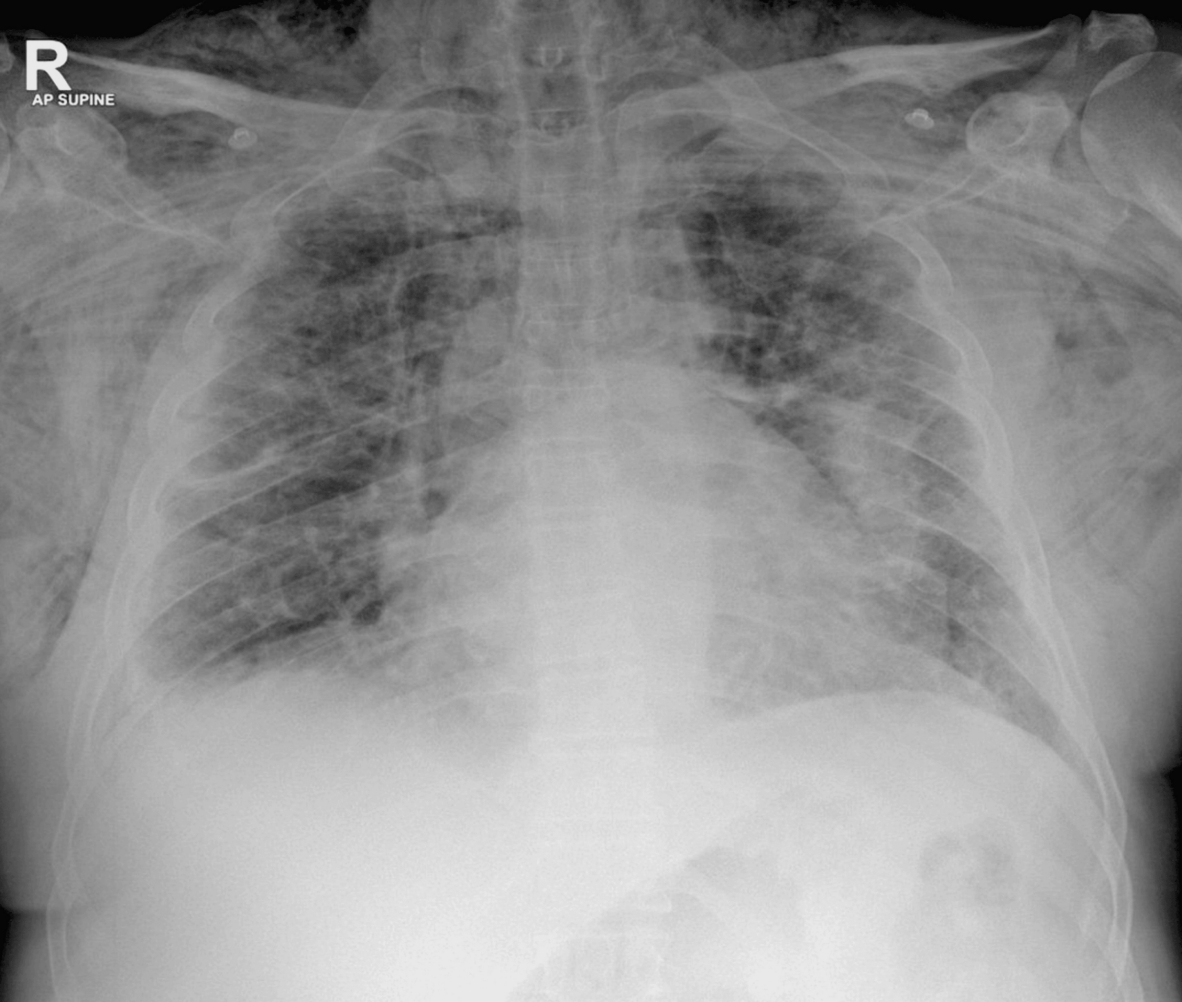
Chest X-ray (AP supine view) demonstrating diffuse subcutaneous emphysema with radiolucent streaks over the thoracic wall.

Two weeks later, the patient presented with progressive swelling of the face, neck, and anterior chest associated with hoarseness and mild dyspnea. On examination, he was hemodynamically stable but mildly tachypneic, with extensive subcutaneous crepitus over the chest wall and neck. Laboratory investigations were unremarkable. Chest radiography demonstrated diffuse subcutaneous emphysema with radiolucent streaks across the thoracic wall, and CT imaging confirmed extensive emphysema extending through the chest wall and soft tissues, as well as pneumomediastinum (Figures 1, 2). 

The patient was initially planned for conservative management and kept under observation for any signs of deterioration. Over the following days, bilateral "blowhole" incisions were performed 3 cm below the clavicles under local anesthesia. The primary team was instructed by the thoracic surgery team to apply wet gauze, partially covering the incisions, and to massage the sites every 3-4 hours. Despite these measures, the patient developed progressive emphysema and acute respiratory distress with stridor, raising concern for impending airway compromise.

The patient was shifted to the emergency OR. Adequate preparations were done in view of an anticipated difficult airway. The ENT team was on standby in case of a "Can't Intubate, Can't Ventilate/Oxygenate (CICV/CICO) situation for emergency tracheostomy. Cardiothoracic and Vascular Surgery teams were also informed. The patient was intubated and ventilated with minimal positive pressure to avoid worsening of the emphysema and subsequently admitted to the intensive care unit. Heliox therapy was suggested by the thoracic team, but it was unavailable in the treating facility. The subsequent day, a 24 Fr subcutaneous drain was inserted into the right chest wall and connected to continuous suction. The patient’s condition improved gradually, and he was successfully extubated after five days. The subcutaneous drain was removed, and he was stepped down from the ICU before being discharged home in stable condition with outpatient follow-up.

## Discussion

SCE following pulmonary resection is a recognized but uncommon complication, with a reported incidence of 1% to 6% after lung resections [1,2]. It results from postoperative air leaks, where air escapes into the mediastinum or subcutaneous tissues through fascial planes. Risk factors include incomplete fissure dissection, fragile lung parenchyma, high airway pressures, prolonged duration of pneumoperitoneum, and forceful coughing post-extubation [4]. In a series of lung resection patients, persistent air leaks were identified as major contributors and advocated early recognition, adequate drainage, and re-exploration when conservative measures fail [1].

Preventive strategies such as intraoperative leak testing, sealant use, and meticulous tissue handling have been emphasized in the literature [2]. Although often self-limiting, severe SCE may progress to airway compromise, tension pneumomediastinum, or impaired venous return, underscoring the importance of early detection [5]. Several decompression methods, including “blowhole” incisions and subcutaneous drains, have been reported, though their effectiveness remains largely anecdotal [3].

Imaging plays a central role in diagnosis, with chest radiography identifying characteristic radiolucent streaks and CT providing definitive confirmation and delineation of the source and extent of emphysema [6]. Since every case is different and hence has to be managed accordingly, the management is based on the overall situation and the surgeon’s preference for treatment, supported by available case reports and expert consensus [7]. Our management of the patient highlights the importance of sharing the clinical expertise and experience of various faculties in Medicine, such as ours. The sequence of events in our patient mirrors previously reported salvage techniques in refractory cases [7,8].

## Conclusions

Extensive SCE is an uncommon but potentially serious postoperative complication following thoracic surgery. While many cases resolve with conservative measures, rapid progression can lead to airway compromise requiring urgent intervention. Our case highlights the importance of early recognition, close monitoring, and prompt escalation of care when standard decompression techniques fail. Multidisciplinary involvement and the use of subcutaneous drainage with continuous suction can be lifesaving in severe cases, underscoring the need for individualized and vigilant postoperative management.
